# Intrapartum Fetal Heart Monitoring Practices in Selected Facilities in Aspirational Districts of Jharkhand, Odisha and Uttarakhand

**DOI:** 10.1007/s13224-020-01403-8

**Published:** 2021-01-17

**Authors:** Enisha Sarin, Devina Bajpayee, Arvind Kumar, Sourav Ghosh Dastidar, Subodh Chandra, Ranjan Panda, Gunjan Taneja, Sachin Gupta, Harish Kumar

**Affiliations:** 1 Monitoring Evaluation and Learning, USAID-VRIDDHI/IPE Global, B-84, Defence Colony, New Delhi, 110024 India; 2 Maternal and Newborn Health, USAID-VRIDDHI/IPE Global, B-84, Defence Colony, New Delhi, 110024 India; 3 USAID-VRIDDHI/IPE Global, B-84, Defence Colony, New Delhi, 110024 India; 4 USAID-VRIDDHI/IPE Global, GVI Campus, RCH Namkum, Ranchi, Jharkhand India; 5 Office of Director General Medical, Health and Family Welfare, Danda Lakhond, Post- Gujrada, Shasrdhara Road, Dehradun, Uttarakhand 248001 India; 6 USAID-VRIDDHI/IPE Global, Bhubaneswar, India; 7 Bhubaneswar, India; 8 USAID, American Embassy, Shantipath, Chanakyapuri, New Delhi, 110021 India

**Keywords:** Fetal heart rate monitoring, Maternal and neonatal health, Fetal ischemia, India, Doppler

## Abstract

**Background:**

The risk of mortality for the mother and the newborn is aggravated during birth in low- and middle-income countries due to preventable causes, which can be addressed with increased quality of care practices. One such practice is intrapartum fetal heart rate (FHR) monitoring, which is crucial for the early detection of fetal ischemia, but is inadequately monitored in low- and middle-income countries. In India, there is currently a lack of sufficient data on FHR monitoring.

**Methods:**

An assessment using facility records, interviews and observation was conducted in seven facilities providing tertiary, secondary or primary level care in aspirational districts of three states. The study sought to investigate the frequency of monitoring, devices used for monitoring and challenges in usage.

**Results:**

FHR was not monitored as per standard protocol. Case sheets revealed 70% of labor was monitored at least once. Only 33% of observed cases were monitored every half hour during active labor, and none were monitored every 5 min during the second stage of labor. More time was observed for monitoring with a Doppler compared with a stethoscope, as providers reported fluctuation in readings. Reportedly, low audibility and a perceived need of expertise were associated with using a stethoscope. High case load and the time required for monitoring were reported as challenges in adhering to standard monitoring protocols.

**Conclusion:**

The introduction of a standardized device and a short refresher training on the World Health Organization and skilled birth attendant protocols for FHR monitoring will improve usage and compliance.

## Introduction

Childbirth is a normal physiological process; however, in low- and middle-income countries (LMICs) there is an increased risk of mortality for the mother and her newborn at the time of birth, due to preventable causes. Over one-third of maternal deaths and life-threatening conditions [1, 2], approximately half of all stillbirths and a quarter of neonatal deaths result from complications during labor and childbirth [3].

India witnesses 32,000 maternal and 640,000 newborn deaths every year [4, 5], and while most of these occur during the period around birth, efforts to improve access to care have not resulted in a concomitant decline in mortality [6], severely calling into question the quality of care provided. Evidence also suggests other persistent causes, such as heavy workloads, which limit the time for history taking, thorough assessments and the provision of timely care; inadequate pre-service and in-service training of health staff; lack of awareness of current recommended effective practices and the availability of skilled personnel; lack of compliance to standard operating procedures; overcrowding at the point of care; and lack of drugs, supplies and equipment to provide effective care [7].

Intrapartum fetal heart rate (FHR) monitoring is crucial for the early detection of fetal ischemia. Prolonged intrapartum fetal ischemia often results in fresh stillbirth or a severely asphyxiated neonate. However, lack of adequate monitoring of FHR is a hallmark of many resource poor settings [8, 9]. The World Health Organization (WHO) recommends listening to the fetal heart rate immediately after a contraction and counting the fetal heart rate for a full minutes at least once every 30 min during the active phase of the first stage of labor and every 5 min during the second stage [10].

A variety of tools and methods used for FHR monitoring in LMIC include stethoscope, Pinard fetal stethoscope, handheld Doppler device or a cardiotocogram (CTG). A Cochrane review concluded that while the use of intermittent CTG and handheld Doppler led to a higher likelihood of the detection of FHR and increased the likelihood of cesarean section for fetal distress compared to a Pinard, there was no difference in perinatal mortality [11]. Pregnant women prefer a hands-on approach to care and are likely to favor any technique that allows this. Service providers prefer to use a Doppler because it offers reassurance and potentially leads to better outcomes for women [12].

Given the inadequate status of monitoring of FHR and the absence of the literature around it in the Indian public health system, the United States Agency for International Development (USAID) *Vriddhi* project undertook an assessment to understand the current situation of FHR monitoring across different levels of public health facilities.

## Methods

### Study Design and Objectives

A mixed methods study, using record review, key informant interviews and observation of labor, was conducted to assess.

- Frequency of FHR monitoring
- Detection level of abnormal FHR
- Devices used and challenges in usage

### Selection of Facilities

The *Vriddhi* project provides technical support to the Government of India’s LaQshya Labor Room Quality Improvement Initiative (Table 1). Three aspirational districts from the states of Jharkhand, Odisha and Uttarakhand were purposively selected for the assessment. A total of seven facilities, three community health centers (CHC), three district hospitals (DH) and a medical college (MC), were assessed on practices and operational challenges. These levels were selected as CHC are the first referral point from a primary healthcare center, district hospitals are the final referral centers for the primary and secondary levels of public health system, and a medical college provides specialized care.

### Selection of Study Participants

Thirty-four service providers per facility was selected purposively for interviews. Thirty-four labor cases per facility were conveniently selected for observation based on the willingness of the pregnant woman and the service provider to participate. Permission was obtained from the National Health Mission, state officials and hospital authorities. Informed consent was obtained from providers and patients before proceeding with observation and interviews. In total, 26 service providers were interviewed, and 27 labor cases were observed. Data collection covered the period from March to June 2019 and was conducted by *Vriddhi* state team members.

### Tools

Case sheets and partographs (wherever available) were reviewed by *Vriddhi* team for monitoring frequency, detection of abnormal FHR and actions taken. The interview questionnaire and the observation checklist, developed by project team, captured the type of device used to monitor FHR, challenges encountered in their use, and whether the staff knowledge staff and the monitoring practice was consistent with WHO standards.

### Data Analysis

The data from records were entered in the project web portal. Previous 4-month data were reviewed for all but two facilities in Odisha, which had collected data for 3 months. The data were compiled in a separate Excel sheet and tabulated for simple frequency and percentages at an aggregate level and facility level. Interview and observation data were likewise entered on Excel sheet.

### Ethical Consideration

The study used data that were publicly available in facility records, and therefore, no ethical clearance was required. Permission was obtained from the Ministry of Health and Family Welfare, the respective state health officials and the facility in charge. Provider interviews and observation of labor cases were conducted only after obtaining written consent from both providers and pregnant women.

## Results

Stethoscope and Doppler were commonly used. CTG was available in the medical college and in two of the district hospitals and used only in a few cases.

### Frequency of Monitoring

Record review highlights that while FHR was monitored at least once in a majority of labor cases (70 percent), it was not frequently monitored after that. About a quarter (27%) were monitored three time and more (Table 2). Due to missing reports, there may be cases where FHR was monitored, but not recorded. The medical college conducted the least amount of monitoring. This may be due to the practice being performed, but not documented because of the very high case load. On observation, it was found that the four cases observed were monitored at 1–1 ½ hours; however, documentation was only for time of admission.

**Table 1:** Facilities across states

State	District hospital	Community health center (CHC)	Medical college
Jharkhand	Chaibasa	Ratu	Rajendra Institute of Medical Sciences (RIMS)
Odisha	Kandhamal	Baliguda	
Uttarakhand	Haridwar	Manglore

**Table 2:** Total frequency of monitoring of FHR according to record review (N = 7310)

No of cases never monitored	559	7.6%
No of cases monitored once	2330	32%
No of cases monitored twice	771	10.6%
No of cases monitored 3–4 times	898	12.3%
No of cases monitored more than 4 times	1124	15.4%
No records available	1628	22.3%

For interviews and observation, monitoring was measured using the standard WHO guidelines for the first and second stages of labor. The table below illustrates this:

A total of 27 labor cases were observed. The observation period varied, lasting from one hour to five hours. Observation was done across stage 1 (*n* = 14) and stage 2 (*n* = 6) labor. Seven additional labor cases were observed across both the stages. A total of 26 service providers were interviewed with questions pertaining to both stages 1 and 2 of labor.

While 69% responded that they monitored FHR every half an hour in stage 1, during observation, only 33% monitored in that frequency (Table 3). There was no reporting or observation of frequent monitoring for the second stage of labor. Observers also examined the case sheets. Many cases were monitored outside the range of standard frequency.

**Table 3:** Frequency of monitoring FHR as per standard guideline (interview and observation data)

Stages of labor	Once every hour	Once every 30 min	Once every 15 min	Once every 5 min	Only at admission	Any other	Missing
Interview (*N* = 26)*							
Stage 1	4	18				4	
Stage 2		3	8	1			
Observation (*N* = 27)							
Stage 1 (n = 14 + 7**)	2	7	0	0	4	7	1
Stage 2 (n = 6 + 7**)		4	2	0	2	4	1

*Interview questions have multiple choice responses, so frequencies do not add up to N

** An additional 7 observers observed both stage 1 and stage 2

Medical college had the lowest percentage (1%) of cases never monitored compared to the other facilities, reflecting that almost every case is monitored. The frequency of FHR monitoring did not account for the time between admission and delivery. Pregnant women access medical colleges from far and often reach in advanced stage of labor. The frequency of FHR monitoring is consequently low. Only one CHC followed correct monitoring for stage 1 (Table 3).

### Reasons for not Complying with Standard Protocol

Reasons given for not monitoring according to standard protocols included high case load (n = 16); too few staff to monitor as required by the guidelines (n = 15); and taking too much time (n = 5).

### Detection of Abnormal FHR

Out of 7310 deliveries, 254 abnormal FHR cases (3.5%) were detected. The majority (63%) of abnormal FHR cases underwent C-section, and 13% were referred out (Table 4). C-sections comprised 22% of the total cases with abnormal FHR responsible for almost 10% of these. While the total still birth and asphyxia rate was 2% and 6%, respectively, the contribution of abnormal FHR to the cases was not recorded.

**Table 4:** Facility level detection of abnormal FHR and action taken

Facility level	Total delivery	Total C-section N(%*)	Total detection of abnormal FHR	C-section of abnormal FHR cases	Referrals of abnormal FHR cases	Assisted delivery of abnormal FHR cases
District hospital	3291	424 (13%)	159 (5%)	87 (55%)	18 (11%)	6 (4%)
CHC	1786	225 (13%)	60 (3%)	22 (37%)	15 (25%)	0
Medical college	2233	976 (44%)	75 (3%)	51 (68%)	0	1 (1%)

*Percentages are rounded

The tertiary facility performed the largest share of C-sections among all facilities, followed by district hospitals. CHCs, on the other hand, referred 25% of abnormal FHR cases. Assisted delivery was low at all levels.

### Use and Reliability of Devices Used

Nearly 77% of the providers reported using a stethoscope, and 58% did so in combination with a handheld Doppler. On observation, an equal number of labor cases were monitored through either a stethoscope or a Doppler (Table 5). The use of CTG was not observed. Interview and observation data show a higher average time to take Doppler readings in contrast to stethoscope readings. Individual readings of different devices could not be separated out when a combination of devices was used.

**Table 5:** Types of device used at facility levels and average time taken for one reading*

Device	Medical college		District hospital		CHC	
	Observation *N* (time taken)	Interview *N* (time taken)	Observation *N* (time taken)	Interview *N* (time taken)	Observation *N* (time taken)	Interview *N* (time taken)
Stethoscope (time taken)	4 (2.5 min)		5 (1.5 min)	4 (2 min)	3 (1.5 min)	2 (1.5 min)
Doppler			6 (3.5 min)	1 (7 min)	7 (4.1 min)	5 (6.5 min)
CTG			1 (5 min)			

*Table does not show combinations of devices

Providers reported difficulty measuring FHR with both stethoscopes and with handheld Doppler. One difficulty related to stethoscope was the time needed to locate a heartbeat. This is contrary to observation findings, which suggests this difficulty is merely a matter of the healthcare staff’s perception. Limited audibility of stethoscope and expertise to use it were other challenges. For Doppler, challenges included difficulty locating the FHR site, fluctuating readings, insufficient numbers as per the total number of daily labor cases, and issues of battery replacement and storage. The Dopplers used in the facilities were of various makes, indicating that there was no one standardized measure in use. With the exception of the district hospital in Odisha, the facilities procured Dopplers locally.

## Discussion

Monitoring did not adhere to standard guidelines. The majority of providers reported monitoring every 30 min; on observation, this standard practice was only seen in 33 percent of the labor cases. This may be due to a social acceptability bias where, during interviews, providers overestimate the frequency of monitoring.

Almost a third of the cases were reported and observed to have been monitored only once at admission and at no other time. Some of the women came to the facility at an advanced stage of labor or immediately before birth, and therefore, there was only time to monitor FHR once. Nevertheless, these frequencies are comparable to those found in a study in Tanzania [12]. In that study, baseline rates of monitoring were extremely low, but, after the implementation of Doppler, monitoring of < 30 min rose to 13 percent of the cases and monitoring between 30–60 min rose to 38 percent, rates already seen in our study. This is perhaps due to the Government of India’s renewed focus on care around birth and the introduction of Doppler in many public health settings in India, which resulted in improved monitoring of FHR. However, the rates in the study still fall far below standard monitoring protocols. Providers maintained that they could not rigorously follow the guidelines due to the high ratio of case load to the number of available staff, a finding echoed in another study [13].

Abnormal FHR was detected in 3–5% of total deliveries in the facilities, slightly higher than what was seen in an African study, where the detection rate of abnormal FHR was 1 percent using a Pinard [12]. In general, in middle-income countries, the detection of abnormal FHR with a Doppler is found to be higher compared to a Pinard [12, 14, 15]. Although health providers reported problems like time needed and low audibility of the stethoscopes, they used them in conjunction with a Doppler.

C-sections were performed on a majority of the cases detected with abnormal FHR. The C-section rate was lower in CHC than in district hospitals and the medical college, while referral was relatively higher, indicating that they need to improve emergency obstetric care. Only one CHC in Jharkhand had the ability perform C-sections. Assisted delivery was low at all levels, as most of the cases ended up delivering by C-section. Abnormal FHR was responsible for 10% of the total C-sections; thus, the additional C-sections could have been elective or performed for other obstetric complications.

Fresh stillbirth rate in the study was two percent of total deliveries. However, it was not documented how many of these were the result of abnormal FHR. There is currently no evidence on positive neonatal outcomes by using one or the other devices [16].

Providers found the Doppler easier to use, but there were issues around locating the site of the FHR, the reliability of the readings, and batteries and storage. Fewer Dopplers were available than required for the case load, resulting in providers relying on stethoscopes. However, the stethoscope had its own problems, as it requires expertise to use. There were also issues around audibility. An interesting point is that while providers believed a stethoscope takes time to auscultate, on observation, they only needed a minute and a half, suggesting their actual proficiency in using the stethoscope. Many of the providers were also seen to fall back on the stethoscope. Perhaps they found it easier to listen to the heartbeat after getting fluctuating readings on the Doppler. Local purchases of Dopplers are not regulated, and hence, there is no guarantee of quality control. It is possible that the use of non-standardized devices made readings confusing and unreliable. In other settings, the Doppler is found to provide reliable readings and thus is well accepted by providers [15, 17]. Indeed, it has been found that handheld devices, including stethoscopes, provide a more hands-on, woman-centered approach to care that is well accepted by both providers and patients [17].

## Limitations

Due to limitations in documentation common in public health facilities, the record review suffers from missing data. It was not possible to exclude pregnant women who presented with full cervical dilatation from the analysis, so cases that required frequent monitoring could not be separated from those that did not. Additionally, the cases of abnormal FHR were not examined individually to detect what specific actions were taken. Results cannot be generalized, as facilities were purposively selected in just three states. However, the district hospital data, CHC data and medical college data can be reasonably seen as representative. Furthermore, observation was not conducted for the entire period of labor, but captured only a segment of the labor. Observation was supplemented by record review preceding and following the observation stage.

## Conclusions

Monitoring of FHR is not performed at the desired frequency in accordance with standard guidelines. This may lead to poor neonatal outcomes, necessitating an intervention to improve compliance. Providers in the study reported challenges in using Dopplers, which may be due to the use of non-standardized equipment. Providers also reported challenges in the use of stethoscopes. The introduction of a standard Doppler which is globally recognized and approved could improve usage and compliance to protocol. Simultaneously, it needs to be tested in order to generate learning. The introduction of such a device is feasible within the given environment of focused intrapartum care. Alongside, a short refresher training or orientation on the WHO and SBA protocol for FHR monitoring would help to reorient labor room staff on the importance and need of frequent FHR monitoring.

## Data Availability

The datasets used and analyzed during the current study are available from the corresponding author on reasonable request.
